# Case Report: Surgery to remove adrenocortical oncocytic carcinoma from an Asian male

**DOI:** 10.3389/fsurg.2023.943296

**Published:** 2023-01-25

**Authors:** Hongtao Liu, Kai Huang, Dan Liu, Yehua Wang

**Affiliations:** ^1^ Department of Urology, General Hospital of Northern Theater Command, Shenyang, China; ^2^ Department of Urology, Clinical Medical College, Yangzhou University, Yangzhou, China; ^3^ Department of Pathology, Clinical Medical College, Yangzhou University, Yangzhou, China

**Keywords:** adrenocortical carcinoma, oncocytic, scoliosis, surgery, case report

## Abstract

Adrenocortical oncocytic carcinoma is a rare type of adrenocortical tumor. Its clinical characteristics and biological behavior need to be further evaluated after the accumulation of cases. Here we report a case of adrenocortical oncocytic carcinoma in an Asian male with scoliosis. We performed an operation on this patient. Because the patient's scoliosis was limited during the operation and the tumor protruded into the chest, we decided to adopt open surgery in the supine position. During the operation, we found a tumor about 8 cm in diameter in the right adrenal region and successfully removed it. The patient recovered well after surgery, and there was no tumor recurrence after one year of follow-up. Pathological results confirmed the diagnosis of adrenocortical oncocytic carcinoma. Pathological features showed tumor cell invasion of adipose tissue, with atypical mitosis and abundant esinophilic cytoplasm. Immunohistochemistry showed that nucleus related antigen (Ki67) index was more than 15% and the positive pathological staining of Synaptophysin (Syn), Melanoma A (Melan A), Inhibin and calretinin. The incidence rate of adrenocortical oncocytic carcinoma is extremely rare. Abdominal Computed tomography (CT) scans and other imaging examination methods are not specific. For larger adrenal tumors, the diagnosis of this disease should be considered. For patients with adrenocortical oncocytic carcinoma who have not yet metastasized, we may achieve sound treatment effects and reduce recurrence by removing the tumor, retroperitoneal fat around the tumor and locoregional lymph nodes.

## Introduction

Adrenocortical oncocytic carcinoma is a rare type of adrenocortical carcinoma (1). Compared with other types of adrenocortical carcinoma, the progression of this kind is relatively slow. Its clinical characteristics and biological behavior need to be further evaluated. In October 2020, on basis of sufficient preoperative preparation, we performed surgery on a patient with an adrenal tumor complicated with scoliosis. Postoperative pathology confirmed that the patient had adrenocortical oncocytic carcinoma, and the treatment effect was satisfactory. The report is as follows.

## Case presentation

**Figure 1:**
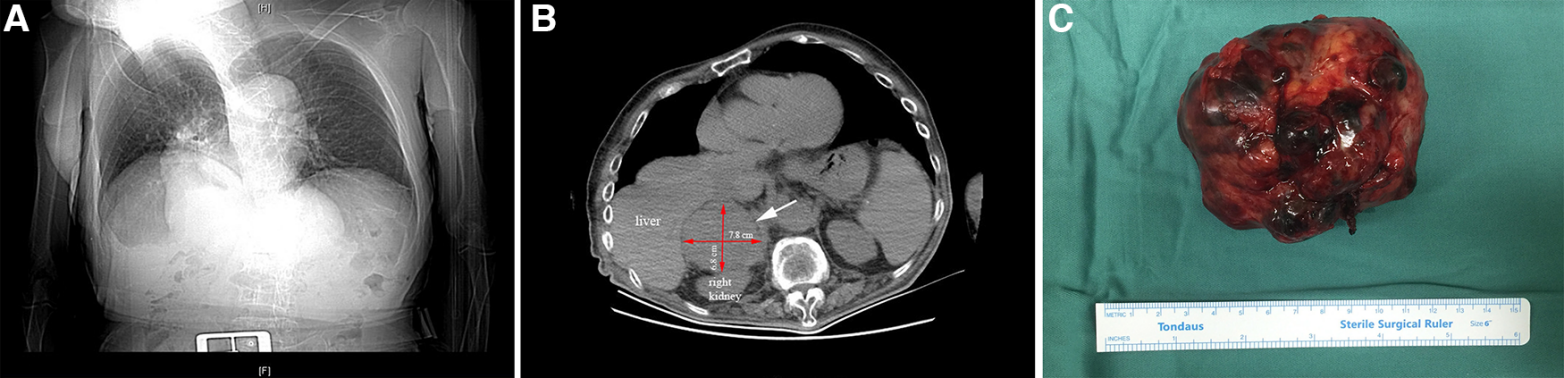
X-ray of chest shows that the patient had scoliosis (**A**). The white arrow points to the location of the tumor. The red arrows indicate the length and width of the mass (**B**). Postoperative mass specimen of adrenocortical oncocytic carcinoma (**C**).

A 72-year-old man came to our hospital for treatment due to the discovery of an adrenal tumor on the right side for more than half a month. The patient had no back pain, hematuria or fever. He had not clinical sign of Cushing's syndrome and had hypertension for 6 years without regular medications and monitoring of blood pressure, but no abnormal palpitation and hypokalemia. His scoliosis was caused by spinal trauma 40 years ago with a Cobb angle reaching 45 degrees (Figure 1A). His blood pressure was 160/96 mmHg when he came to our hospital. An abdominal CT scan showed a tumor in the right adrenal area measuring 78 mm × 68 mm (Figure 1B). The boundary of the mass was clear with homogeneous enhancement. There was no local invasion or lymph node enlargement. Preoperative examination showed that the urinary vanillylmandelic acid level was 17.5 mg/24 h (hr) (reference value range 0∼12 mg/24 h). Serum aldosterone and cortisol levels were normal. Before the operation, doxazosin was used to control blood pressure for 2 weeks. After his blood pressure was maintained at around 140/90 mmHg and his heart rate was stable at around 90 beats/min, an operation was performed.

Because the patient's scoliosis limited the surgical approach from the back and the tumor protruded into the chest, we decided to adopt open surgery in the supine position. An incision was made under the costal margin, and the surrounding organs were pushed aside. The tumor was found in the right adrenal region. There was local adhesion with the surrounding tissues. The surface blood vessels of the tumor were distended. After the blood vessels around the tumor were ligated and removed, the tumor was completely removed (Figure 1C). The blood loss was approximately 300 ml, and the highest intraoperative blood pressure was 182/110 mmHg.

**Figure 2:**
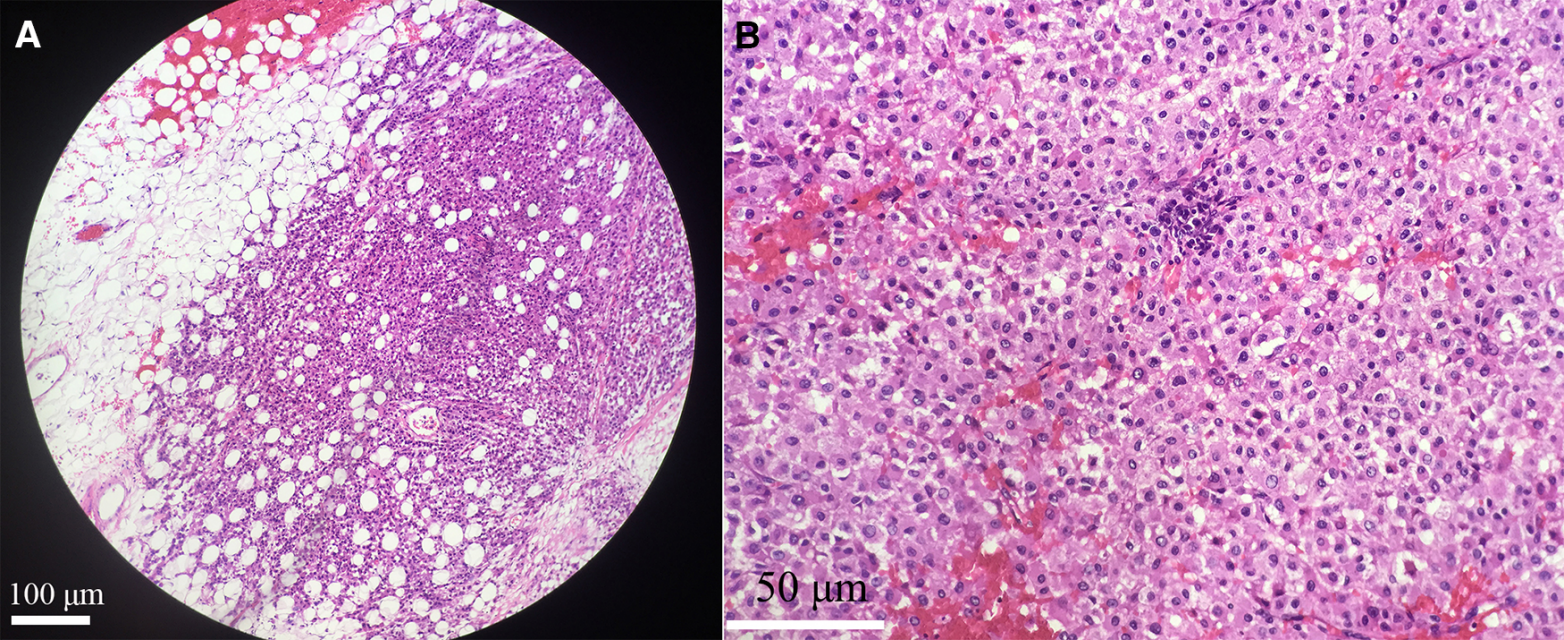
The tumor cells invaded adipose tissue, original amplification ×100 (**A**). There were obvious stromal vessels with necrosis in tumor tissue, original amplification ×400 (**B**).

**Figure 3:**
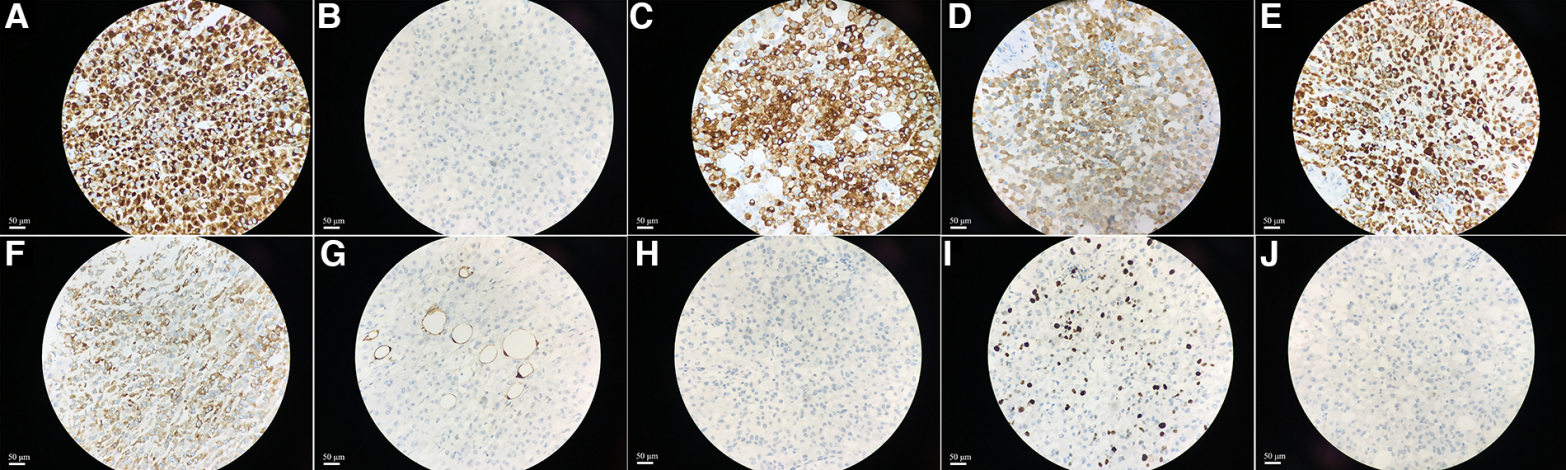
The immunohistochemical staining showed the expression of Vimentin, NSE, Syn, Inhibin, Melan A, calretinin, S-100, Desmin, Ki67 and Alk in tissues, original amplification ×400 (**A–J**).

Postoperative pathological results were as follows (Figure 2): the tumor cells were arranged in nests with heteromorphic cells; there were obvious nucleoli, atypical mitosis, abundant eosinophilic cytoplasm; and the tumor cells invaded the adipose tissue and had obvious stromal blood vessels. Immunohistochemistry showed the following (Figure 3): vimentin (+), Neuron specific enolase (NSE) (−), Syn (+), Inhibin (+), Melan A (+), calretinin (+), S-lfln protein 100 (S-100) (−), Desmin (−), Ki67 (approximately 15%+) and Anaplastic Lymphoma Kinase (Alk) (−). No lymph node metastasis was found. The pathological diagnosis was adrenocortical oncocytic carcinoma. The patient was rated as 3 points according to the Weiss score system.

After one year of follow-up, the patient recovered well without disease recurrence.

## Discussion

Adrenocortical carcinoma is a rare malignant tumor, with an incidence rate of only 1 to 4 persons every 2 million, and includes the oncocytic, mucinous, sarcomatous and other tissue types (2). Patients with adrenocortical carcinoma have a poor prognosis and are prone to relapse and metastasis. The median overall survival duration is only 3 to 4 years. Patients with adrenocortical oncocytic carcinoma tend to have larger tumors, a lower recurrence rate and a better prognosis than those with other subtypes (3).

Most patients with adrenocortical carcinoma have no obvious symptoms. When the tumor grows gradually, they may have low back pain. Approximately 30% of adrenocortical oncocytic tumors secrete hormones, which may lead to hypercortisolism and personality abnormalities.

Imaging examinations are of limited value in the diagnosis of adrenocortical oncocytic carcinoma. CT scans and Magnetic resonance imaging (MRI) are the commonly used examination methods. Recently, 18Fluoro-Fluorodeoxyglucose (F-FDG) Positron Emission Tomography (PET)/CT was used in the diagnosis of adrenocortical carcinoma. PET/CT showed that the uptake of FDG in the tumor area was intense but showed inhomogeneous enhancement, while there was no uptake in the adrenal hyperplasia area. This may be related to the overexpression of Glucose transporter1 (GLUT1) and hexokinase II in the tumor area (4).

The Weiss scoring system is the most important method to differentiate benign and malignant adrenocortical tumors (5). The main criteria include mitosis greater than 5/50 High power field (HPF), atypical mitosis, and venous invasion. The secondary criteria include tumor diameter >10 cm and weight >200 g, tissue necrosis, capsular and blood sinus invasion. If it meets one of the primary criteria, the tumor can be diagnosed as malignant. If only one or more secondary criteria are met, it can be considered potentially malignant. If it does not conform to any of the main criteria or secondary criteria, the tumor can be considered benign. In this report, pathological imaging showed that mitosis was more than 5/50 HPF and tumor cells invaded the adipose tissue. Therefore, the diagnosis was adrenocortical oncocytic carcinoma. Duregon evaluated 225 patients with adrenocortical carcinoma and found that patients with higher Weiss scores had worse prognoses (2).

A high Ki67 index is associated with tumor growth and tissue differentiation ability. For patients with adrenocortical oncocytic tumors, the Ki67 index is very important for diagnosis and prognosis (6). Pathologic results of this patient showed a Ki67 index greater than 15%, which was consistent with the manifestation of adrenocortical carcinoma. Advani et al. (7) thought that an elevated Ki-67 index is a feature of adrenocortical carcinoma, although it does not appear to predict individual patient survival. The immunohistochemical profile of adrenocortical carcinoma was positive for Melan A and calretinin (8). Zlatibor et al. (9) reported that the average survival rate and median duration of survival in the group of adrenocortical carcinoma patients with positive staining for Melan A was eight times longer than patients with negative. Four times as patients had positive inhibin staining than negative. The negative immunohistochemical staining of Syn and Inhibin is often related to the poor prognosis of patients with adrenocortical carcinoma (9). This patient may have a low chance of recurrence because of the positive pathological staining of Syn and Inhibin. It has also been confirmed that patients with adrenocortical oncocytic carcinoma have better prognoses than those with other subtypes (3).

Surgery is still the main method for localized adrenocortical oncocytic carcinoma. Retroperitoneal laparoscopic surgery or open surgery through the 11th intercostal incision in the lumbar back can be used. In this case, due to the large volume of the tumor and scoliosis with a Cobb angle of 45 degree, we adopted an open operation with a subcostal incision to ensure good visual exposure. During the operation, we removed the tumor and its surrounding retroperitoneal fat as whole. Meanwhile, we performed local lymph node dissection to ensure the resection scope. Although the patient had scoliosis and a thoracic deformity, we still gently pushed the liver, diaphragm, intestine and other surrounding organs to acquire good vision. This ensured the successful resection of the tumor.

For patients with recurrence risk after resection, mitotane can be used as adjuvant therapy (10). Radiotherapy, radiofrequency ablation, and chemoembolization are of special value for patients with advanced adrenocortical carcinoma who cannot undergo operation. Researchers have gradually realized the relationship between molecular changes in signal pathways such as Insulin-like growth factor 2 (IGF2), Wingless Integrated (Wnt)/*β*-catenin, Tumor Antigen P53 (TP53), Retinoblastoma (Rb) and the occurrence, development, recurrence, metastasis, and therapeutic effect of adrenocortical carcinoma (11). Some molecular targeted drugs have been developed. Some researchers applied a humanized mouse model of adrenocortical carcinoma to explore the effect of anti-programmed cell death protein 1 (PD1) immunotherapy on the tumor microenvironment (12). The results showed that the experimental group showed obvious tumor growth inhibition, and the tumor did not easily metastasize.

In conclusions, the incidence rate of adrenocortical oncocytic carcinoma is extremely rare. CT scans and other imaging examination methods are not specific. For large adrenal tumors, the diagnosis of this disease should be considered. Surgical treatment should be the first choice after diagnosis. It is necessary to have regular follow-ups after the operation. The limitation of our study is the relatively small number of patients with adrenocortical oncocytic carcinoma. According to few reports, adrenocortical oncocytic carcinoma seem to have a better prognosis than other types of adrenocortical carcinoma. We need to further evaluate the characteristics and biological manifestations of adrenocortical oncocytic carcinoma through more cases.

## Data Availability

The original contributions presented in the study are included in the article/Supplementary Material, further inquiries can be directed to the corresponding author/s.
